# Effect of Targeted Yoga Practice on PMS Symptoms, Body Composition, and Hip Mobility: An Eight-Week Pilot Study

**DOI:** 10.3390/sports14010021

**Published:** 2026-01-05

**Authors:** Vanda Agnes Nemes, Eszter Mikó-Baráth, Charlotte Briest, Dorottya Szabó, Tibor Mintál, Balázs Patczai, Katalin Gőcze

**Affiliations:** 1 Department of Traumatology and Hand Surgery, Clinical Centre, University of Pécs, Ifjúság Str. 13, H-7624 Pécs, Hungary; vanda.nemes@aok.pte.hu (V.A.N.); patczai.balazs@pte.hu (B.P.); 2 Institute of Physiology, Medical School, University of Pécs, Szigeti Str. 12, H-7624 Pécs, Hungary; 3 Sports Medicine Center, Medical School, University of Pécs, Akac Str. 1, H-7632 Pécs, Hungary; 4 Pathology Innovation Incubator Ltd., Bokay Janos 36-42, H-1083 Budapest, Hungary; krgocze@gmail.com

**Keywords:** premenstrual syndrome, yoga intervention, body composition, hip mobility, women’s health

## Abstract

Premenstrual syndrome (PMS) negatively affects women’s physical performance, emotional balance, and quality of life. Although pharmacological therapies exist, their side effects and limited effectiveness highlight the need for alternatives. This partially controlled, non-blinded, non-randomized prospective pilot study included 34 women aged 18–40 years and examined the effects of an eight-week structured yoga program. Participants met the same eligibility criteria and were comparable at baseline in age, sociodemographic characteristics, and PMS severity. The study group attended two weekly 90-min hatha yoga sessions and completed a 15-min daily home practice, while controls maintained their usual physical activity. Outcome measures included body composition, hip range of motion, spinal mobility (flexion and lateral flexion), and Premenstrual Symptoms Screening Tool (PSST) scores. After eight weeks, the yoga group showed significant reductions in body weight and fat mass and an increase in muscle mass. Hip external rotation improved significantly among yoga participants, while changes in spinal mobility did not reach statistical significance. The intervention group showed a significant reduction in PMS symptom severity, while the control group showed no significant change. The findings suggest that regular yoga practice can enhance physical functioning and alleviate PMS-related symptoms, supporting its role as a movement-based approach for improving women’s health.

## 1. Introduction

Premenstrual syndrome (PMS) is characterized by recurrent psychological and physical symptoms during the luteal phase of the menstrual cycle that resolve spontaneously within the first few days of menstruation. While up to about 80% of women in reproductive age experience some symptoms, approximately 5% report severity that interferes with daily functioning, relationships, and work performance [1,2,3].

PMS commonly includes affective symptoms such as mood swings, depression, irritability, anxiety, fatigue, and reduced concentration, alongside physical issues such as nausea, sleep problems, breast tenderness, weight gain, abdominal pain, and skin changes [1,4,5]. These can cause distress, lower well-being, job satisfaction, and productivity, and can affect performance in mentally or physically demanding professions.

The American College of Obstetricians and Gynecologists (ACOG) recommends supporting the diagnosis with validated tools like the Premenstrual Symptoms Screening Tool (PSST) in combination with detailed symptom tracking (e.g., Daily Record of Severity of Problems, DRSP) over at least two menstrual cycles [1,2,5,6,7]. The PSST on its own is also suitable for screening purposes and follow-up measurements.

Although the exact cause of PMS and PMDD is unclear, symptoms are closely linked to hormonal fluctuations and increased CNS sensitivity, with proposed mechanisms involving neurotransmitter imbalance, neuroendocrine disturbances, and stress-related factors [8,9,10]. Recent evidence suggests that both musculoskeletal factors and body composition may influence the severity of somatic complaints during PMS [11,12,13,14]. Women with menstrual pain often show postural alterations, pelvic and spinal misalignment, and reduced mobility, contributing to increased musculoskeletal tension and lower back pain [14,15]. Correcting spinal alignment and pelvic asymmetries has been shown to alleviate menstrual pain and discomfort [13,14,15]. Higher body mass and fat percentage have been also linked to more severe PMS symptoms [11,12].

PMS management strategies range from lifestyle changes and patient education to pharmacotherapy, in which case long-term adherence is limited by adverse effects [2,10]. Therefore, there is growing interest in complementary approaches, including yoga, for its combined physical and psychological benefits [2,16,17,18].

Yoga, rooted in ancient Indian philosophy, combines physical postures (asanas) with breath control (pranayama) and meditation or mindfulness techniques. Its effectiveness in alleviating PMS symptoms is supported by a growing body of evidence highlighting its psychological and physical benefits [16,19,20,21,22]). These studies have largely focused on psychological or subjective symptom outcomes measured via standardized tools such as the PSST, DRSP, and other scales [16,19,20], and only a few have explored how structured programs may improve functional parameters, such as body composition or joint mobility, alongside symptom relief. This is a notable omission, given that many women with PMS report somatic symptoms that may have biomechanical underpinnings. Although dysmenorrhea-focused studies have begun to explore the impact of targeted stretching, spinal mobility, and core-strengthening yoga poses on pain and function, such dimensions are rarely addressed in PMS trials [8,21]. Moreover, while some research suggests that yoga can improve weight and body composition in women with PMS, few studies integrate these physical health markers alongside symptom tracking [22,23]. This lack of multidimensional assessment limits our understanding of how yoga may influence both subjective and physiological aspects of PMS [9,16,24,25]. Unlike exercise-only or mindfulness-based interventions, yoga integrates physical conditioning with relaxation techniques, providing a more comprehensive approach that can influence both musculoskeletal function and symptom perception [16,26].

### Aims of the Study

Previous PMS-related yoga studies vary widely in style, duration, and outcome measures, and most rely primarily on subjective symptom reporting, rarely incorporating objective physical health indicators [16,19,27]. To address this gap, the present study examined whether an eight-week structured yoga intervention could produce measurable changes in body composition, hip and spinal mobility, and PMS symptom severity in women of reproductive age.

This selection of core physical outcomes was based on emerging evidence showing that restricted mobility in the lumbar–pelvic region and increased musculoskeletal tension may exacerbate somatic symptoms in women. Although this area is less studied in PMS specifically, available research shows that improved mobility and reduced regional tension may also contribute to alleviating PMS-related discomfort. Given the limited research directly addressing this link, integrating ROM assessments represents a novel contribution to this area of research.

Accordingly, the aim of the study was to evaluate whether a targeted yoga protocol, incorporating hip- and spine-focused postures, breathing exercises, and guided relaxation, would lead to improvements in both subjective PMS symptom burden and objective musculoskeletal function, compared with a control group maintaining habitual activity levels. PMS symptoms were assessed using the validated PSST, while physical outcomes were evaluated through standardized body composition and joint mobility measurements.

We hypothesized that participants completing the eight-week yoga program would show (1) measurable short-term improvements in body composition and hip and spinal mobility and (2) significant reduction in PMS symptom severity.

## 2. Materials and Methods

### 2.1. Participants

Participants were recruited via university mailing lists, posters, and social media platforms targeting female staff and students at the University of Pécs, Hungary. The study was advertised as a free and voluntary opportunity to participate in research for women who subjectively felt that PMS affected their daily lives. Recruitment aimed to target women of the same socioeconomic background. Interested volunteers contacted the research team via email, and pre-screening was carried out based on the inclusion and exclusion criteria. Inclusion criteria involved the willingness to participate, the ability to consent, and an age of 18–40 years. Exclusion criteria included pregnancy, childbirth within the past year, the presence of serious chronic or endocrine disorders, and regular yoga practice (a minimum of once weekly) within the previous 6 months. After subject eligibility was confirmed, a total of 34 female volunteers were enrolled in the program. All participants received written and oral information about the aims and course of the study and provided their written informed consent. The women who decided to participate in the yoga program served as the intervention group (*n* = 19, mean age 27.59 ± 4.04 SD), and those who could not adhere to the regular program were selected as the control group (*n* = 15, mean age 30.61 ± 5.51 SD). The study was approved by the Regional and Local Research Ethics Committee at the University of Pécs (ethics approval number: 6655) and conducted in accordance with the Declaration of Helsinki as revised in October 2008.

### 2.2. Procedure and Study Design

The study followed a prospective, partially controlled, non-blinded, non-randomized design. The intervention group attended the eight-week yoga program that included two 90-min-long guided group classes per week, complemented with a 15-min-long daily home practice. The women in the control group continued their usual lifestyle without any structured physical activity. In the control group, PSST questionnaire assessment was the only measurement performed; body composition and anthropometric measurements were not conducted.

### 2.3. The Yoga Program

The yoga sessions were led by a certified yoga instructor who was also a medical research student on this project (Author CB). The yoga protocol was developed in collaboration with a certified yoga therapist, and the asanas were chosen according to their assumed beneficial effects upon the female organs as well as on the hormonal system. Specific focus was on the hip and back region, pelvic floor, and breathing and relaxation practices (Table 1).

The classes started with the sun salutations (Suriya Namaskar) for warming up (15 min) and continued with a series of various yoga postures for 60 min, which always included the specifically chosen series of asanas: squatting pose (Kagasana), butterfly pose (Bhadrasana), half bow pose (Utthita Dhanurasana), and candle pose (Sarvangasana). The postures were followed by a 5-min-long alternate nostril breathing exercise (Nadi Sodhana) and finished with a 10-min guided relaxation (Savasana). Each class included brief awareness phases between postures to promote self-observation and enhance body–mind connection and deeper relaxation. To maintain variety and engagement, additional asanas were also incorporated into the sessions in a consistent manner (once a week each). These included forward bends (sitting and standing), spinal twists (sitting and lying), balance poses (eagle), and inversions, in addition to warm-up exercises and sun salutations. Progression was implemented by gradually increasing posture duration and by enhancing difficulty through added muscular engagement (e.g., breath-synchronized pelvic floor activation and posture complexity). All sessions were supervised and followed a standardized structure to ensure reproducibility and held in university-provided facilities.

The additional short daily home practice consisted of two specific asanas, squatting pose and butterfly pose, alongside alternate nostril breathing, totalling approximately 15 min per day.

Adherence to both the yoga classes and home practice was monitored, allowing a maximum of 20% absence (up to three missed sessions) over the eight-week program. To accommodate participants’ schedules, three classes per week were offered.

### 2.4. Measures

#### 2.4.1. Body Composition Measurements

In the case of the intervention group, body composition was evaluated with bioelectrical impedance analysis (BIA, OMRON BF 511, Omron Healthcare Inc., Muko, Japan) at the beginning and the end of the yoga program. Parameters of interest were body weight, body mass index (BMI), fat content, muscle mass, and visceral fat percentage.

#### 2.4.2. Joint Mobility

Joint mobility was evaluated pre- and post-intervention by a certified physiotherapist among members of the study group. Spinal mobility was assessed in a standing position through measurements of trunk flexion (forward bend) and trunk lateral flexion (side bend), using fingertip-to-floor distance measured in centimeters [28]. Hip range of motion (ROM) was assessed in the supine position with knees flexed to 90°. Passive abduction and outward and inward hip rotation were assessed using a goniometer [29].

#### 2.4.3. PMS Symptoms

PMS symptom severity was assessed based on the validated self-reported Premenstrual Symptoms Screening Tool (PSST) [7,30]. This questionnaire is designed to evaluate both the presence and impact of physical, emotional, and behavioral symptoms associated with the luteal phase. Both the intervention and control groups completed the questionnaire at baseline and after eight weeks. The PSST has demonstrated strong psychometric properties in reproductive-age women, with high internal consistency, good test–retest reliability, and robust convergent validity [30,31,32]. These validation studies were conducted in populations above the age of 18, supporting the suitability of the PSST for our sample.

#### 2.4.4. Feedback Forms

Participant feedback was collected anonymously at the end of the intervention using an in-house-developed questionnaire. The form included eight items rated on a 10-point Likert scale (1 = very poor, 10 = excellent) assessing overall experience, organization of the study, home practice, yoga classes, and the instructor. In addition, participants could provide open-ended written comments regarding their personal experiences and suggestions for improvements.

### 2.5. Data Analysis

Statistical analysis was carried out with MedCalc for Windows, version 20.211 (MedCalc Software Ltd., Ostend, Belgium). Data were analyzed with the paired samples *t*-test after confirming normal distribution of the data with Kolmogorov–Smirnov test. For the comparison of PSST outcomes on the 0–3 scale, Wilcoxon and Mann–Whitney tests were used for the within- and between-groups comparisons, respectively. In case of missing data (i.e., one participant did not attend any of the post-intervention evaluations), the participant was completely excluded from that analysis, and a lower number of cases were reported for that subsection (data considered Missing Completely at Random).

## 3. Results

### 3.1. Body Composition Measurements

A significant change was observed in the following parameters: decreases in mean body weight and body fat content and an increase in muscle mass (Table 2). A nonsignificant decrease was found in Body Mass Index (BMI) and the proportion of visceral fat. In these anthropometric outcomes, although several “before–after” differences reached statistical significance, the corresponding Cohen’s d values were small, indicating that the magnitude of change was modest despite the low *p*-values.

### 3.2. Joint Movement Ranges

As 3 out of the 19 participants did not attend either the before or the after joint movement anthropometric measurements, their data were completely excluded from this statistical chapter, and only the data of 16 participants were included in this analysis. Flexibility of the spine was assessed in two directions by measuring finger–floor distance in centimeters during forward bend and side bends (lateral flexion). In the yoga group, forward bend performance, quantified as finger–floor distance, showed a small, nonsignificant improvement from baseline (4.38 cm ± 6.73 SD) to post-intervention (2.75 cm, ±6.89 SD). Using finger–floor distance was a possible limiting factor because it cannot capture further gains in participants who were able to reach the floor, as improvements beyond this point could not be quantified under the standardized protocol. In the case of lateral flexion, the distance remained unchanged at 43.44 cm (±6.33 SD) on a group level (both sides together).

The mean hip ROM in external rotation was 25.78° (±6.49 SD) before the study and increased to 28.44° (±4.48 SD, *p* = 0.024) after the eight weeks of yoga. Inward rotation measures showed an average ROM of 33.91° (±5.64 SD) initially and 34.16° (±6.29 SD) with a *p* = 0.78 after the study. Abduction decreased, with both sides grouped together on average with 48.13° (±6.19 SD) before the yogic intervention and 47.50° (±5.68 SD) after the program (*p* = 0.65) (Table 3).

### 3.3. Premenstrual Symptom Severity

In the case of the intervention and the control groups, altogether 18 and 15 participants filled in the PSST questionnaires, respectively, both at the beginning and at the end of the study.

The PSST score characterizes the severity of the symptoms on a four-grade score using 18 criteria (Total Symptom Severity Score). An additional five items (A–E) evaluate functional impairment domains (Total Functional Impairment Score). Grading was carried out according to the criteria of Steiner et al. [7,30], which define moderate to severe PMS as follows: (1) at least one of the core affective symptoms (items #1–4) is rated as moderate to severe; (2) at least four of the fourteen symptoms overall (items #1–14) are rated as moderate to severe; and (3) at least one of the functional impairment domains (A–E) is rated as moderate to severe. Based on these criteria, 67% of the intervention group and 60% of the control group met the threshold for moderate to severe PMS at baseline. Following the intervention, this proportion decreased to 50% in the intervention group, while remaining unchanged (60%) in the control group (Table 4).

Overall, statistical analysis with the Wilcoxon test on paired samples showed significant within-group improvements for the following items of the PSST: anxiety (*p* = 0.034), difficulty concentrating (*p* = 0.021), fatigue (*p* = 0.046), hypersomnia (*p* = 0.017), physical symptoms (breast tenderness, pain, bloating, etc., *p* = 0.012), and work efficiency (*p* = 0.029) in the yoga group, and there was no significant change in the control group.

The mean PSST total score in the intervention group (max. score: 57) decreased from 18.06 (±7.99 SD) to 13.00 (±7.23 SD) (*p* = 0.006). The score for the psychological and physical symptoms (Total Symptom Severity Score, max. score: 42) also significantly (*p* = 0.004) decreased from 14.39 (±6.38 SD) to 10.50 (±5.29 SD). Total Functional Impairment Score (max. score: 15) also decreased from 3.67 (±2.89 SD) to 2.50 (±2.28 SD), although this change was nonsignificant (*p* = 0.151) (Table 5).

In contrast, the control group showed no significant change, with baseline mean PSST total scores of 16.27 (±9.34 SD) and 15.60 (±9.49 SD), pre- and post-study, respectively (*p* = 0.73). Total Symptom Severity Score was 13.67 (±7.83 SD) at the beginning and 12.47 (±7.08 SD) at the end of the study (*p* = 0.44), and Total Functional Impairment Score increased from 2.60 (±2.35 SD) to 3.13 (±3.25 SD) with *p* = 0.39 (Table 5). Even though the standard deviations are overall very large, the tendencies are still apparent in the data.

### 3.4. Feedback Form

Participants rated the program very positively, with an overall evaluation of 9.69 (±0.63 SD) on the 1–10-point Likert scale. Guided yoga classes were rated 9.54 (±0.78 SD), and the instructor received the highest score (9.92 ± 0.28 SD). The organization of the study was rated 9.08 (±1.32 SD). Lower ratings were given for scheduling (8.15 ± 1.52 SD) and location (university sports hall) (8.69 ± 1.70 SD), and daily home practice, which was considered the most challenging aspect, was rated 7.92 (±1.61 SD).

## 4. Discussion

Several studies support the efficacy of yoga for PMS relief. Notably, even brief interventions (e.g., 4 weeks) can improve mood and sleep disturbances, while longer programs appear to yield broader symptom reductions, including pain and bloating [16,19,27,33]. However, most prior research has relied mainly on subjective symptom-based assessments and has seldom included physical outcome measures such as musculoskeletal function or body composition.

The eight-week structured yoga program resulted in significant improvements in body composition, a significant reduction in PSST symptom severity, and significant gains in hip outward ROM. These findings demonstrate that a targeted yoga intervention, combining pelvic-focused asanas, breathing techniques, and guided relaxation, can produce measurable benefits across physical, functional, and symptom-related parameters.

### 4.1. Body Composition

In the current study, the eight-week yoga program led to measurable and statistically significant improvements in body composition, comparable to outcomes of other short-term, exercise-based interventions in women [34]. The reduction in body fat percentage (−1.09%, *p* = 0.012) and the increase in muscle mass (0.56 kg, *p* = 0.006) are notable given the relatively brief duration of the intervention. Similar reductions in body fat have been documented in yoga-based programs [35,36], and increases in lean mass have been reported following longer-term ashtanga practice [37]. These are within-group intervention-related changes rather than definitive comparative effects. Nevertheless, the results suggest that the program provided sufficient muscular stimulus, likely through the sustained isometric loading of postures and the dynamic sequences of sun salutations to elicit favorable body composition changes.

Differences between our findings and earlier studies reporting no change in body composition may relate to the characteristics of the intervention [38,39]. Several prior yoga programs emphasized relaxation or low-intensity practices, whereas the present protocol combined dynamic flows with sustained isometric holds and incorporated both supervised sessions and daily home practice, potentially producing a higher cumulative training load. Participant characteristics, such as being yoga-naïve, may also contribute to differential responsiveness.

Body composition was assessed using bioelectrical impedance analysis (BIA). Although BIA can be influenced by many factors, it is widely used in clinical and exercise research and shows good agreement with DXA when evaluating group-level, intervention-related mean changes [40,41]. As our study focused on group-level differences rather than individual diagnostic accuracy, BIA was considered an appropriate and accessible measurement tool. Nevertheless, the modest sample size limits the precision of the estimates, and the observed changes, although statistically significant, should be interpreted as preliminary.

### 4.2. Hip and Spinal Flexibility

Passive hip range of motion (ROM), including abduction and external and internal rotation, was measured in degrees before and after the yoga intervention. The core postures in the program were selected to increase ROM, with an emphasis on abduction and external rotation to enhance pelvic mobility, release myofascial tightness around the hip, and improve neuromuscular control [38]. Consistent with exercise-specific adaptation principles, some within-group intervention-related changes were found. The most notable improvement occurred in external rotation, in alignment with prior research showing that hip-opening postures, such as Baddha Konasana and Kagasana, effectively mobilize the external hip rotators [39,42,43,44]. These findings parallel other studies reporting flexibility gains in the pelvic and lumbar regions following yoga practice [44,45]. In contrast, inward rotation showed minimal change and hip abduction no change, even though these movement ranges were also intended targets of the intervention. This may reflect a combination of factors, such as the greater emphasis of the selected postures on external rotation rather than pure frontal-plane abduction, illustrating that flexibility improvements are greatest in the ranges that are deliberately trained [39,46]. The practical implication of this finding is that comprehensive, balanced sequences are needed in future studies when the goal is multi-planar hip mobility. Other possible explanations could be limited baseline restriction in abduction ROM, measurement technique, and the possibility that an eight-week intervention was insufficient to elicit measurable group-level changes in this movement plane.

Given that hatha yoga inherently targets hip opening, spinal mobility, neuromuscular relaxation, and postural balance, range of motion (ROM) change represents a plausible and responsive functional indicator of the intervention’s physical effects. Assessing hip and spinal mobility, therefore, allowed us to explore whether improvements in musculoskeletal function accompany changes in PMS symptoms, an aspect that remains underexamined in prior yoga-related PMS studies. Our long-term aim is to position ROM assessments as functionally relevant, objective markers that complement subjective symptom scoring and help bridge an existing gap in PMS intervention research.

Although most research on hip and spinal mobility relates to dysmenorrhea rather than PMS, many PMS symptoms, such as pelvic discomfort, low back tension, and a sense of heaviness, originate from the same pelvic and lumbar region. This suggests that targeted hip-opening and mobility-focused practices may also contribute to relief of PMS-related somatic symptoms through improved regional mobility and altered ascending proprioceptive feedback [38,47,48,49]. This potential pathway remains underexplored in PMS research, and the present study provides preliminary evidence supporting the relevance of musculoskeletal factors in PMS management.

### 4.3. Premenstrual Symptoms Screening Tool

The eight-week yoga program, focusing on hip mobility, pelvic floor muscles, relaxation, and breathing techniques, resulted in significant improvements in PSST scores within the intervention group, while no change was detected in the control group. The most frequently reported complaints—anxiety, difficulty concentrating, fatigue, hypersomnia, physical symptoms (breast tenderness, pain, bloating, etc.) and work efficiency—showed significant within-group improvements according to a Wilcoxon test on paired samples, aligning with previous findings that yoga can reduce both physical and psychological PMS manifestations [16,33,50]. The relatively large standard deviations likely reflect the heterogeneous baseline PMS severity within the sample, which is characteristic of PMS populations [19]. Unlike many relaxation-focused programs, this intervention used targeted asanas selected for PMS-related somatic complaints, achieving benefits comparable to longer protocols [22,33,51,52].

Analysis of PSST categories also showed fewer participants remaining in the mild-to-moderate range post-intervention, indicating clinically meaningful reductions in both the number and severity of symptoms [16,33,50].

We acknowledge that, neither on the level of single items (1–15, A–D) or in the merged PMDD scores emerged significant between-group differences post-intervention; this likely reflects the limited sample size, as power calculations suggest that larger cohorts would be required to detect modest group-level effects on the independent samples. The control group also completed only questionnaire-based assessments and did not participate in any structured activity, which constrains firm conclusions about the yoga-specific nature of the observed improvements. Together with the inherent cycle-to-cycle variability of PMS symptoms, these factors may have limited the detection of more consistent group-level effects.

While the PSST is a validated and widely used screening tool, it relies on retrospective recall and may introduce memory-related variability. More detailed prospective symptom-tracking tools (e.g., DRSP) were not used because pre and post assessments could not be aligned to individual menstrual cycle phases; the group-based design with a fixed starting date made cycle-phase-specific scheduling logistically and methodologically infeasible. This limitation, along with the absence of daily symptom diaries, is acknowledged.

Despite these constraints, the combination of validated symptom measures and functional mobility outcomes provides a more comprehensive view of yoga’s potential benefits. Improvements in hip external rotation and spinal mobility may reduce regional tension and enhance ascending proprioceptive feedback, offering a plausible physical contribution to symptom relief. Further randomized, adequately powered studies with active control groups, cycle-aligned assessments, and prospective symptom tracking are needed to confirm these findings and to clarify the relationship between pelvic mobility and affective PMS symptoms.

Overall, the results align with prior yoga research documenting reductions in both physical and psychological PMS manifestations [16,33,50].

We also acknowledge that, neither on the level of single items (1–15, A–D) or in the merged PMDD scores, emerged significant (according to Mann–Whitney test) between-group differences post-intervention; this likely reflects the limited sample size, as power calculations suggest that larger cohorts would be required to detect modest group-level effects on the independent samples.

Participant feedback indicated that the study design, two weekly classes combined with daily home practice, was overall feasible within their daily routines. While some challenges were noted, particularly regarding scheduling and adherence to home practice, the high satisfaction ratings suggest good acceptability of the program. These insights provide valuable guidance for refining the setup and organization of future yoga interventions.

### 4.4. Limitations

Despite the promising results, some limitations should be acknowledged and considered when interpreting the findings.

The study included a modest, non-randomized sample. In addition, the control group participated only in questionnaire-based assessments and did not undergo anthropometric or mobility testing, restricting direct between-group comparisons to subjective outcomes. The study was underpowered to detect moderate differences in joint range of motion and PSST symptom scores, as the achieved sample size was substantially lower than the 30–40 (within group) and 60–70 (between-group) participants estimated to be required. Consequently, some potentially clinically relevant effects may not have reached statistical significance, particularly in the between-group analysis of PSST. Although several variables showed significant within-group differences, the observed effect sizes were generally small to moderate, reflecting the exploratory nature of this pilot study and suggesting that larger, well-powered samples would be needed to confirm the clinical relevance of these preliminary results.

The study also employed a non-blinded design, and participants were aware of their group allocation; as in most exercise-based interventions, blinding was not feasible, and this may have introduced expectation-related bias. A convenience sampling strategy and self-selection into study groups may have introduced selection bias, as participants who were more health-conscious, motivated, or interested in yoga may have been more likely to enrol in the intervention.

PMS symptoms and body weight can vary across menstrual cycles, and although all participants followed the same fixed study timeline, measurements were not aligned to individual cycle phases, which may have added variability. However, intervention effects were only evaluated on a group level to account for this limitation. The reliance on the retrospective PSST questionnaire, without daily prospective symptom tracking, may also have introduced recall bias.

Finally, the eight-week duration and the recruitment of volunteers from a university population limit generalizability, and the study was not designed to isolate yoga-specific mechanisms. These limitations highlight the need for larger, randomized, and longer-term studies incorporating phase-aligned assessments, objective physiological measures, and active control conditions to strengthen future conclusions.

## 5. Conclusions

To summarize, this study demonstrates that an eight-week structured yoga program can reduce PMS symptom severity while also improving body composition and selected aspects of musculoskeletal function, particularly hip external rotation. Even though the primary hypothesis regarding spinal mobility and hip abduction was unsupported in this short pilot study, the findings add to the growing evidence that yoga, through its combined strength, flexibility, and relaxation components, can alleviate both physical and psychological manifestations of PMS. The integration of validated symptom measures with objective functional outcomes provides a comprehensive understanding of yoga’s therapeutic potential, and the feasibility of the home practice component indicates that such programs could be incorporated into routine health promotion settings.

Given its low cost, accessibility, and favorable acceptability ratings, the yoga protocol used in this study may serve as a practical adjunctive option for women seeking non-pharmacological approaches to managing PMS symptoms. Such programs could be implemented in community or university health settings, workplace wellness initiatives, or primary care-based lifestyle counselling. However, larger and longer-term studies are needed before formal clinical recommendations can be established.

Due to the modest sample size and the absence of between-group significance, these results should be interpreted as preliminary. Future randomized, adequately powered studies—ideally incorporating cycle-aligned assessments, active control groups, prospective symptom tracking, and more detailed biomechanical measures—are required to confirm these effects and further clarify the role of pelvic mobility in PMS symptom improvement. A longer-term aim is to replicate these findings in larger samples and explore whether anthropometric variables (e.g., via ROC analysis) can help distinguish between milder and more severe PMS presentations.

## Figures and Tables

**Table 1:** Overview of the yoga intervention protocol and baseline characteristics of participants.

Element	Description			
Instructor	Certified yoga instructor (medical research student, Author CB)			
Focus areas	Hip region, pelvic floor, breathing, relaxation			
Class structure (90 min)	- 15 min warm-up: Surya Namaskar (sun salutations) - 60 min postures (Kagasana, Bhadrasana, Utthita Dhanurasana, Sarvangasana) - 5 min alternate nostril breathing (Nadi Shodhana) - 10 min guided relaxation (Savasana)			
Additional elements	Awareness phases between postures, progressive increase in duration and intensity, pelvic floor training integrated into Bhadrasana			
Home practice	Daily, 15 min: Kagasana, Bhadrasana, Nadi Shodhana			
Adherence	≤20% absence allowed Three weekly time slots (afternoon/evening) offered			
Outcome measures	Changes in body composition Hip joint and spine mobility PSST questionnaire			
Participants	Parameter (±SD)	Intervention group (*n* = 19)	Control group (*n* = 15)	Comparison
	Age	27.59 ± 4.04	30.61 ± 5.51	Comparable
	Having child (yes)	21.05%	26.67%	Comparable
	Education level (high school vs. university degree)	31.57%	33.33%	Comparable
	Total Symptom Severity Baseline	14.39 ± 6.38	13.67 ± 7.83	N.s.d. (*p* = 0.77)
	Total Functional Impairment Baseline	3.67 ± 2.89	2.60 ± 2.35	N.s.d. (*p* = 0.26)
	Total PSST Score Baseline	18.06 ± 8.00	16.27 ± 9.34	N.s.d. (*p* = 0.56)

The results are expressed in percentage or as Mean ± SD. Baseline PSSD scores were compared by independent samples *t*-test. N.s.d.: no significant difference.

**Table 2:** Body composition data of the members of the intervention group (n = 19) before and after the eight-week yoga program.

Parameter	Before	After	*p*-Value	Cohen’s d
Body weight (kg)	64.12 ± 10.97	63.36 ± 11.40	**0.021**	0.068
BMI (kg/m^2^)	22.55 ± 3.19	22.37 ± 3.26	0.247	0.055
Body fat (%)	31.67 ± 6.93	30.58 ± 7.59	**0.012**	0.149
Muscle mass (%)	28.63 ± 2.84	29.19 ± 3.15	**0.006**	0.188
Visceral fat (%)	3.95 ± 1.27	3.74 ± 1.28	0.104	0.165

‘Before’: at the start of the intervention, ‘After’: after the completion of the eight-week yoga program. Data are presented as mean ± standard deviation (SD). *p*-values in bold refer to significant change (*p* < 0.05) based on paired samples *t*-test. BMI: Body Mass Index.

**Table 3:** Joint movement ranges in the members of the intervention group (*n* = 16) before and after the eight-week yoga program.

Hip Movement (R + L)	Before	After Mean	*p*-Value	Cohen’s d
Forward bend	4.38 ±6.73	2.75 ± 6.89	0.053	0.24
Side bend	43.31 ± 5.53	43.44 ± 5.10	0.84	0.02
Hip Abduction	48.13° ± 6.19	47.50° ± 5.68	0.65	0.13
Outward Rotation	25.78° ± 6.49	28.44° ± 4.48	**0.024**	0.47
Inward Rotation	33.91° ± 5.64	34.16° ± 6.29	0.78	0.042

‘Before’: at the start of the intervention, ‘After’: after the completion of the eight-week yoga pro-gram. Data are presented as mean ± standard deviation (SD). *p*-values in bold refer to significant change (*p* < 0.05) based on paired samples *t*-test.

**Table 4:** Percentage of participants meeting criteria for moderate to severe PMS before and after intervention, according to the scoring method of Steiner [7,30].

Criteria Met for Moderate to Severe PMS	Intervention Group (*n* = 18)		Control Group (*n* = 15)	
	Before (%)	After (%)	Before (%)	After (%)
1.	67	50	60	60
2.	55	22	60	53
3.	28	11	20	27

‘Before’: at the start of the intervention, ‘After’: after the completion of the eight-week yoga program. Definitions of PMS according to criteria 1–3 are described in the body of the text.

**Table 5:** Premenstrual Symptoms Screening Tool (PSST) scores.

	Intervention Group (*n* = 18)			Control Group (*n* = 15)		
PSST Score	Before	After	*p*-Value	Before	After	*p*-Value
Total Symptom Severity	14.39 ± 6.38	10.50 ± 5.29	***p* = 0.004**	13.67 ± 7.83	12.47 ± 7.08	*p* = 0.44
Total Functional Impairment	3.67 ± 2.89	2.50 ± 2.28	*p* = 0.15	2.60 ± 2.35	3.13 ± 3.25	*p* = 0.39
Total PSST Score	18.06 ± 8.00	13.00 ± 7.23	***p* = 0.006**	16.27 ± 9.34	15.60 ± 9.49	*p* = 0.73

The results are expressed as Mean ± SD. Scores for symptom severity, functional impairment, and total score are presented separately for the study (*n* = 18) and control (*n* = 15) groups before and after the eight-week interval. Statistically significant differences (*p* < 0.05) are shown in bold.

## Data Availability

The datasets used and analyzed during the current study are available from the corresponding author on reasonable request.

## Abbreviations

The following abbreviations are used in this manuscript:

ACOG	American College of Obstetricians and Gynecologists
BIA	Bioelectrical Impedance Analysis
BMI	Body Mass Index
CNS	Central Nervous System
DRSP	Daily Record of Severity of Problems
HPA	Hypothalamic-Pituitary-Adrenal
PMDD	Premenstrual Dysphoric Disorder
PMS	Premenstrual Syndrome
PSST	Premenstrual Symptoms Screening Tool
ROM	Range of Motion
SD	Standard Deviation
